# Delivering hypertension care in private-sector clinics of urban slum areas of India: the Mumbai Hypertension Project

**DOI:** 10.1038/s41371-022-00754-1

**Published:** 2022-09-24

**Authors:** Asha Hegde, Haresh Patel, Chinmay Laxmeshwar, Ajit Phalake, Anupam Khungar Pathni, Ravdeep Gandhi, Andrew E. Moran, Mandar Kannure, Bhawana Sharma, Vaishnavi Jondhale, Sapna Surendran, Shibu Vijayan

**Affiliations:** 1 PATH, Mumbai, India; 2 Resolve to Save Lives, New Delhi, India; 3 Resolve to Save Lives, New York, NY USA; 4 Columbia University Irving Medical Centre, New York, NY USA

**Keywords:** Hypertension, Health care

## Abstract

In India, the private sector provides 70% of the total outpatient medical care. This study describes the Mumbai Hypertension Project, which aimed to deliver a standard hypertension management package in private sector clinics situated in urban slums. The project was conducted in two wards (one “lean” and one “intensive”) with 82 private providers in each. All hypertensive patients received free drug vouchers, baseline serum creatinine, adherence support, self-management counseling and follow-up calls. In the intensive-ward, project supported hub agents facilitated uptake of services. A total of 13,184 hypertensive patients were registered from January 2019 to February 2020. Baseline blood pressure (BP) control rates were higher in the intensive-ward (30%) compared with the lean-ward (13%). During the 14-month project period, 6752 (51%) patients followed-up, with participants in the intensive-ward more likely to follow-up (aOR: 2.31; *p* < 0.001). By project end, the 3–6-month cohort control rate changed little from baseline—29% for intensive ward and 14% for lean ward. Among those who followed up, proportion with controlled BP increased 13 percentage points in the intensive ward and 16 percentage points in the lean ward; median time to BP control was 97 days in the intensive-ward and 153 days in lean-ward (*p* < 0.001). Despite multiple quality-improvement interventions in Mumbai private sector clinics, loss to follow-up remained high, and BP control rates only improved in patients who followed up; but did not improve overall. Only with new systems to organize and incentivize patient follow-up will the Indian private sector contribute to achieving national hypertension control goals.

## Background

Uncontrolled blood pressure (BP) leads to more than ten million deaths annually which are half the total cardiovascular deaths globally [1]. In the past decades, hypertension has become prevalent among the populations of low and middle-income countries, which account for about 80% of people living with hypertension worldwide [2, 3]. According to 2019 estimates, hypertension is among the most common non-communicable diseases in India, with a prevalence of 31.6% among men and 30.5% among women in the age group of 30–79 years [3]. Among those treated, 17.3% men and 18.5% women are estimated to have their BP under control [3].

The World Health Organization (WHO) has set a global target of a 25% reduction in hypertension prevalence by 2025 [4]. In 2017, the Ministry of Health and Family Welfare, Government of India, adopted a national action plan for prevention and control of non-communicable diseases with an aim to achieve 25% reduction in mortality from cardiovascular diseases, and 25% reduction in hypertension prevalence by 2025 [4].

The India Hypertension Control Initiative (IHCI), also launched in 2017, aims to strengthen the management of hypertension in the public health sector and provides a continuum of care by strengthening treatment and follow-up of patients toward BP control [5]. The IHCI has five intervention strategies based on the WHO HEARTS technical package—(1) standard drug- and dose treatment protocol, (2) uninterrupted supply of drugs, (3) team-based care and task sharing, (4) patient-centered services by enabling availability of BP monitoring and drug refills closer to the patients, and (5) monitoring system to track individual patients’ treatment and BP control [6].

Whilst the IHCI is expanding in public sector facilities across the country, it is estimated that more than 70% of outpatient visits in India occur in the private health care sector [7]. There is a significant unmet need for hypertension treatment in the country [8], a gap that will only be filled by engaging the private sector in the campaign to achieve the country’s NCD goals. The Mumbai Hypertension Project was implemented by PATH and was designed to deliver the WHO HEARTS-IHCI hypertension management package in private sector primary care clinics situated in the urban slums of Mumbai, the principal city of Maharashtra state. In two wards of Mumbai, a service delivery model was designed to ensure people living with hypertension receive standardized treatment according to the state protocol, support, treatment adherence, and monitor and document individual and program progress toward improved hypertension control. Additional services were offered in one of the two wards (intensive ward) while offering limited support in the other ward (lean ward). We hypothesized that the more intensive hypertension management services package in the intervention ward would lead to better cohort BP control and patient follow-up rates compared to the lean ward.

## Methods

### Study design and setting

The study was conducted in two wards of Mumbai, India. Mumbai is a metropolis of 21 million people, with nearly half the population living in slums [9]. The prevalence of hypertension in Mumbai is estimated to be 25.9% for adults above 18 years of age [10]. Populations in Mumbai slums are reported to have poor access to diagnosis and underdiagnosis of non-communicable diseases [11, 12]. PATH, a global non-profit organization, had worked with private service providers in the slums of Mumbai through its public private interface agency model to ensure early and accurate diagnosis of tuberculosis, effective case management and successful treatment for patients through universal access to quality services [13]. Detailed description of this model is available elsewhere [14, 15]. The current hypertension control study leveraged PATH’s experience in working with the private providers in Mumbai.

After conducting a scoping exercise in six wards of Mumbai, two wards were selected for the project (Supplementary Fig. 1). The first ward, G-North, was a dense slum cluster with a population of 602,238. The second ward, the N-ward, had sporadic slum clusters and a population of 622,594.

**Fig. 1:**
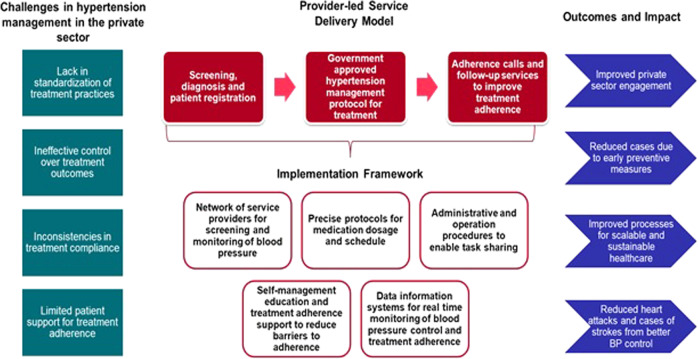
Conceptual framework of the Mumbai hypertension project. The framework describes the underlying challenges in hypertension management in the private sector, the service delivery model, the implementation framework, and the expected outcomes.

The project conceptual framework was designed to address anticipated challenges to the successful management of hypertension in India’s private sector. A service delivery model was developed with three key interventions as seen in Fig. 1. The project was designed as an iterative model to enable quick operational changes and its adaptation to project activities to maximize impact. The project on-boarded private-sector service providers (doctors, pharmacists and laboratories) in both the wards.

### Provider selection

We established a network of private practitioners in the study area who reported diagnosing more than ten hypertensive patients a month at baseline and were willing to comply with the study protocols. These included practitioners with Doctor of Medicine (MD), Bachelor of Medicine and Surgery (MBBS) and Ayurveda, Homeopathy and Unani degrees (together termed as AYUSH providers). All practitioners in the study received training on hypertension management according to the study protocols. A validated digital sphygmomanometer was provided to all practitioners. The frequency of follow-up visits depended on the number of patients being enrolled by the practitioners—those who enrolled fewer patients were visited more often. A total of 82 practitioners were enrolled in each ward over the course of the project. Among these, in the lean ward, 60 (73%) were AYUSH, 10 (12%) were MBBS and 12 (15%) were MD providers. In the intensive ward, 44 (54%) were AYUSH, 18 (22%) were MBBS and 20 (24%) were MD providers. Engagement of the private providers in the lean and intensive wards is depicted in Supplementary Fig. 2. Additionally, follow-up activities with empaneled private providers to ensure adherence to protocol are depicted in Supplementary Fig. 3.

Participants were enrolled in the study from January 2019 till February 2020. However, due to the COVID-19 pandemic, follow-ups could not be conducted regularly from February 2020 and the study procedures were stopped. While the project activities continued till February 2020, post November 2019, project activities were gradually phased out.

### Project interventions

**Fig. 2:**
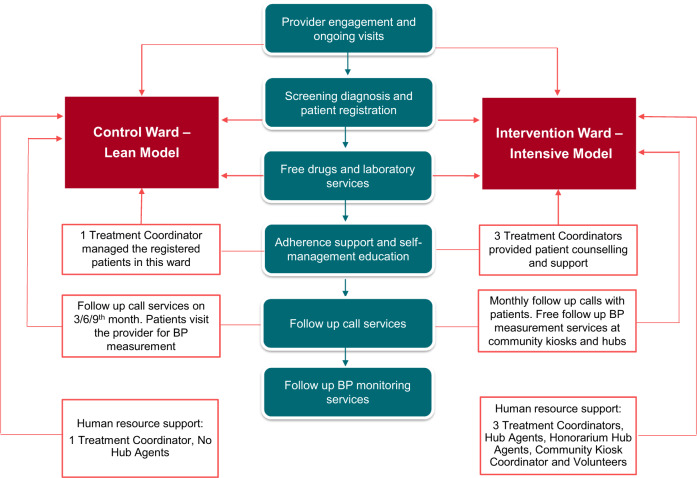
Intervention models in lean and intensive wards in the Mumbai hypertension control project. This figure illustrates the interventions offered in the lean and intensive models.

Different intervention models were implemented in the two wards as shown in Fig. 2.

In both the wards, hypertensive patients willing to register under the project received project services such as free drug vouchers, free serum creatinine test, adherence support, self-management counseling and follow-up call services to encourage patients to regularly follow-up with their service provider. The duration of the vouchers was coterminous with the next follow-up date. In addition, in the intensive ward, hub agents were placed in selected facilities with high patient load to improve patient registrations and to support service providers in facilitating uptake of services offered under the project. Some hub agents were full time project staff, while others were clinic staff and were provided an honorarium to conduct additional duties for the project. Of the 82 providers in the intensive ward, 16 facilities were provided project hub agents, while 14 were provided honorarium hub agents. The remaining 52 facilities in the intensive ward did not have any hub agents. Hub agents were given a target for enrollment based on the records and were eligible for incentives if they exceeded those targets.

Along with the hub agents, the project also provided for treatment coordinators. The treatment coordinator would call the patient 48 h after enrollment to provide lifestyle management and adherence counseling. The treatment coordinator would also contact all patients who missed their appointments. In the intensive ward, three treatment coordinators were provided to conduct adherence and monthly follow-up calls to registered patients, whereas, in the lean ward, only one treatment coordinator conducted adherence and quarterly follow-up calls.

Based on the performance of the project sites, over the course of the project, several modifications were made to the project operations. The changes included prioritization of field visits to providers based on their performance and potential to deliver targets, incentivization of hub agents for patient registration, simplification of data collection tools, introduction of fixed dose combinations (FDCs) to increase project hypertension protocol drug prescriptions and voucher utilization, intensive follow-up call week, call center services, and setting up of community kiosks for BP measurements. The FDCs available through the project included Telmisartan 40 mg and Amlodipine 5 mg, Telmisartan 40 mg and Chlorthalidone 6.25 mg, Telmisartan 40 mg and Hydrochlorothiazide 12.5 mg, Amlodipine 5 mg and Telmisartan 40 mg and Chlorthalidone 6.25 mg. In addition, some strategies that did not work were discontinued. The mid-course corrections and their effect on patient enrollment is shown in Supplementary Fig. 4.

### Study participants

Patients above the age of 18 years and diagnosed with hypertension were enrolled after written consent was provided. Hypertension was defined as systolic BP ≥ 140 mmHg or diastolic BP ≥ 90 mmHg on two successive occasions. Patients who were known hypertensives were also included. Patients with secondary hypertension were excluded.

### Data collection and analysis

Details of all patients enrolled in the study were entered into a patient card by the practitioners or their staff. Patients were provided lab vouchers for serum creatinine testing and antihypertensive drug vouchers as required. The counterfoils of the patient card, lab voucher and drug vouchers were collected from the empaneled clinics, laboratories and pharmacists respectively on a regular basis by the project field staff and compiled. All data was regularly monitored by the study staff for accuracy and completeness.

The primary outcome was the difference between a change in cohort hypertension BP control rates between the intensive and lean wards. Cohort BP control was based on an indicator of hypertension program performance as recommended in the WHO HEARTS technical package [16]. Cohort control was assessed for each wave of enrolled participants (cohorts) 3–6 months after they were initiated on treatment under the project. For 3–6 monthly cohort BP control rates, analysis was conducted using data of (1) all patients registered; and (2) only those patients who followed up in the quarter.

The secondary outcome was the difference in change in follow-up rates between the two wards. Follow-up rates were analyzed for patients with at least one follow-up visit 1-month after enrollment under the project. Other indicators that were analyzed were the proportion of patients with uncontrolled BP at baseline who achieved control and time to BP control. Kaplan–Meier curves were used to describe the time to BP control among patients with at least one follow-up visit. The cohort enrolled till December 2019 was considered for logistic regression and survival analysis.

Data were analyzed using R. Categorical variables among demographic and clinical variables are summarized using numbers and proportions, and continuous variables are described using the median and interquartile range. Logistic regression controlling for the ward, age, gender, voucher utilization, hub agent, provider type, and follow-up phone calls was used to describe the factors associated with controlled BP and age, gender, voucher utilization, hub agent, provider type, BP measurement at registration, proportion of follow-up phone calls, proportion of follow-up visits, duration of treatment, and ward for patient follow-up (at least once). Irrespective of the number of follow-up visits, the last available BP measurement was taken as final for this analysis.

## Results

### Patient characteristics

A total of 13,184 hypertensive patients were registered from January 2019 to February 2020. The baseline demographic and clinical characteristics are shown in Table 1. The median age was 53 years (IQR: 45–62 years) with almost half being males. The median systolic BP was 150 mmHg (IQR: 136–162) and diastolic BP was 90 mmHg (IQR: 80–100). Baseline proportion with controlled BP was higher in the Intensive ward (30.0%) compared with the Lean ward (12.9%).

**Table 1:** Demographic and clinical characteristics of hypertensive patients enrolled in the Mumbai Hypertension Project.

	Lean ward	Intensive ward	Total
Patients registered *n*	4560	8624	13,184
Median age in years (IQR)	50 (42–60)	54 (45–62)	53 (45–62)
Age categories *n* (%)			
<30 years	157 (1.8)	121 (1.4)	278 (2.1)
30–44 years	1232 (27.0)	1665 (19.3)	2897 (22.0)
45–59 years	1845 (40.5)	3795 (44.0)	5640 (42.8)
60+ years	1326 (29.1)	3043 (35.3)	4369 (33.1)
Sex *n* (%)			
Male	2257 (49.5)	4042 (46.9)	6299 (47.8)
Female	2303 (50.5)	4582 (53.1)	6885 (52.2)
Mean baseline blood pressure (SD)			
Systolic	155.1 (20.1)	147.7 (22.7)	150.3 (22.1)
Diastolic	93.2 (11.5)	88.4 (13.5)	90.1 (13.1)
Baseline blood pressure control *n* (%)			
Stage 1^a^	1448 (31.8)	2779 (32.2)	4227 (32.1)
Stage 2^b^	2521 (55.2)	3257 (37.8)	5778 (43.8)
Within normal range	591 (13.0)	2588 (30.0)	3179 (24.1)
Median follow-up time in months (IQR)^c^	0.9 (0–5.6)	2.4 (0–5.8)	2.0 (0–5.8)
Median number of follow-upvisits (IQR)^c^	1 (0–4)	1 (0–4)	1 (0–4)

^a^Defined as systolic blood pressure between 140–159 mm of Hg and diastolic blood pressure between 90–99 mm of Hg and is uncontrolled BP.

^b^Defined as systolic blood pressure more than 159 mm of Hg and diastolic blood pressure more than 99 mm of Hg and is uncontrolled BP.

^c^For cohort enrolled till September 2019.

### Trend and provider patterns in hypertensive patient enrollments over project period

There was a steady increase in the new registrations trend every month from January 2019 until November 2019. AYUSH providers registered the maximum number of patients in both the wards. Overall, 59% patients were registered by AYUSH providers, while MD and MBBS service providers registered 23% and 18% patients respectively. On average, there were 76 patient registrations per AYUSH provider, 86 patient registrations per MBBS provider, and 134 patient registrations per MD provider. Overall, 37% total patient registrations were from the 16 facilities where project hub agents were placed, 17% from the 14 facilities with honorarium hub agents, and 46% patient registrations from facilities with no hub agents (134 facilities).

### Patient follow-up

Out of the 13,184 hypertensive patients enrolled in the project, 6752 patients (51.3%) followed up at least once with their service provider during the project period (Supplementary Fig. 5). In the lean ward, 2348 (51.5%) patients never followed-up, while this number was 4084 (47.4%) in the intensive ward. Due to the impact of the COVID-19 pandemic, 824 patients registered in January–February 2020 did not receive follow-up calls and were excluded from the analysis.

After adjustment for baseline characteristics, participants in the intensive ward were more likely to follow-up than those in the lean ward (aOR: 1.64; 95% CI: 1.41–1.91) (Table 2). Participants enrolled with providers with the presence of a project hub agent (aOR: 1.61; 95% CI:1.37–1.90) or honorarium hub agent (aOR: 1.28; 95% CI: 1.08–1.52) were more likely to return for follow-ups compared to providers where no hub agents were present. Among providers, participants registered with MD providers were less likely to follow-up (aOR: 0.32; 95% CI: 0.28–0.37), while those with MBBS providers were more likely to follow-up (aOR: 1.22; 95% CI: 1.08–1.39) compared to AYUSH providers.

**Table 2:** Factors associated with at least one patient follow-up visit in the Mumbai hypertension project.

	Adjusted odds ratio^a^	95% confidence interval	*p* value
Ward			
Lean	Ref		
Intensive	1.64	1.41–1.91	<0.001
Age			
18–44 years	Ref		
45–59 years	1.27	1.14–1.42	<0.001
≥60 years	1.08	1.00–1.29	0.04
Gender			
Male	Ref		
Female	1.19	1.08–1.31	<0.001
Voucher utilization			
No	Ref		
Yes	3.61	3.20–4.08	<0.001
Hub agent			
No hub agent	Ref		
Project hub agent	1.61	1.37–1.90	<0.001
Honorarium hub agent	1.28	1.08–1.52	<0.001
Provider type			
AYUSH	Ref		
MBBS	1.22	1.08–1.39	<0.001
MD	0.32	0.28–0.37	<0.001
Follow-up phone calls			
Not connected	Ref		
Connected	2.76	2.46–3.09	<0.001

^a^Odds ratios from logistic regression models adjusting for ward, age, gender, voucher utilization, hub agent, provider type, and follow-up phone calls.

### Blood pressure control

Quarterly cohort BP control rates at 3–6 months after enrollment among all registered patients and among those who followed up was better in the intensive ward as compared to the lean ward throughout the study (Table 3a, b). Among all participants, by the time the third cohort of hypertension patients was followed up (those registered between July-September 2019), the cohort control rate had improved by ten percentage points in the intensive ward and four percentage points in the lean ward (Table 3a). However, the improvement in cohort control decayed by the time of the fourth cohort (those registered October–December 2019), such that there was no clear evidence that study interventions and modifications over time to reduce loss-to-follow-up translated to improved cohort control. Among only those participants with at least one follow-up visit, compared with the first cohort of hypertension patients, cohort control in both the intensive and lean wards improved in the second and third cohorts, then decayed by the time of the fourth and final cohort (Table 3b).

**Table 3:** Cohort blood pressure control achieved by 3–6 months after enrollment.

a. 3–6 monthly cohort BP control rate of all patients enrolled under the Mumbai Hypertension Project							
**Quarter of enrollment**		**Lean**		**Intensive**		**Total**	
		**# of Pts enrolled**	**3–6 months BP control of all enrolled patients**	**# of Pts enrolled**	**3–6 months BP control of all enrolled patients**	**# of Pts enrolled**	**3–6 months BP control of all enrolled patients**
Q1 (Jan–Mar 2019)		457	11%	572	20%	1029	16%
Q2 (Apr–Jun 2019)		634	18%	1338	23%	1972	21%
Q3 (Jul–Sept 2019)		1462	15%	2609	30%	4071	24%
Q4 (Oct–Dec 2019)		1421	14%	3174	17%	4595	16%
Total		3974	14%	7693	22%	11667	20%
b. 3–6 monthly cohort control rate of only the patients enrolled under the Mumbai Hypertension Project who followed up in the next quarter							
**Quarter of enrollment**	**Lean ward**			**Intensive ward**			**Total**
	**# of Pts enrolled**	**# of Patients followed up in next quarter**	**3–6 months BP control of those who followed up**	**# of Pts enrolled**	**# of Patients followed up in next quarter**	**3–6 months BP control of those who followed up**	**3–6 months BP control of those who followed up**
Q1 (Jan–Mar 2019)	457	257	20%	572	350	32%	27%
Q2 (Apr–Jun 2019)	634	351	32%	1338	769	40%	38%
Q3 (Jul–Sept 2019)	1462	709	31%	2609	1561	50%	44%
Q4 (Oct–Dec 2019)	1421	656	29%	3174	1341	39%	36%
Total	3974	1973	29%	7693	4021	43%	38%

Because the proportion with controlled BP was higher in the intensive ward at baseline, BP control was also compared among only participants with uncontrolled BP at baseline. Of the 13,184 patients enrolled under the project, 10,005 patients had uncontrolled BP at baseline. In the lean ward, of the 3969 patients with uncontrolled BP at baseline, 12.8% achieved BP control on the last recorded follow-up visit. Correspondingly, in the intensive ward, of the 6036 patients with uncontrolled BP at baseline, 25.6% achieved BP control on the last recorded follow-up visit (Supplementary Fig. 5).

### Time to blood pressure control among patients retained in care

**Fig. 3:**
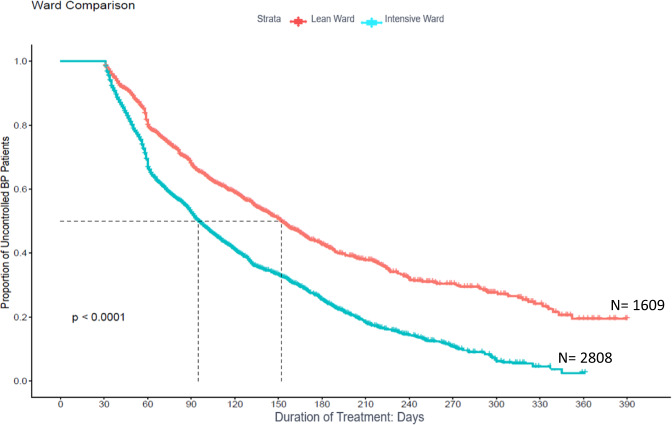
Kaplan–Meier curve describing the time to blood pressure control in intensive and lean wards in the Mumbai Hypertension Project. This figure shows the time to BP control patients whose BP was uncontrolled at the time of registration and who came for follow-up.

In the time to BP control analysis conducted for 4417 patients whose BP was uncontrolled at the time of registration and who came for follow-up (Fig. 3), the estimated median time taken for controlling BP was 113 days (3.7 months). There was a significant difference in the median time to BP control between the lean and the intensive wards (log rank test *p* < 0.001). In the intensive ward, BP was controlled in 97 days (3.2 months), while in the lean ward, the time to attain BP control was 153 days (5.1 months).

### Factors associated with blood pressure control among those followed up

Logistic regression showed that after adjusting for “gender, voucher utilization, hub agent, provider type, BP measurement at registration, age, proportion of follow-up phone calls, proportion of follow-up visits, and duration of treatment” patients in the intensive ward (aOR: 2.31; *p* < 0.001) were more likely to have BP under control then lean ward (Table 4). Compared to those with an age less than 45 years, patients in the higher age groups showed better BP control. The qualification of the provider also affected BP control, with patients following up with MBBS (aOR: 1.48; *p* < 0.001) and MD (aOR: 1.41; *p* < 0.001) providers showing better control than those visiting AYUSH providers.

**Table 4:** Factors associated with blood pressure control in the Mumbai Hypertension Project.

	Total (*N* = 5995)		Intensive ward (*N* = 4022)		Lean ward (*N* = 1973)	
	Odds ratio	*p* value	Odds ratio^a^	*p* value	Odds ratio^a^	*p* value
Gender						
Male	Ref					
Female	–	–	0.77	0.00	0.75	0.00
Voucher utilized						
No	Ref					
Yes	–	–	0.64	0.00	0.93	0.65
Hub agent						
No hub agent	Ref					
Project hub agent	–	–	1.18	0.10	–	–
Honorarium hub agent	–	–	2.19	0.00	–	–
Provider type						
AYUSH	Ref					
MBBS	–	–	1.48	0.00	1.24	0.13
MD	–	–	1.41	0.00	1.81	0.01
BP measurement at registration						
Controlled BP	Ref					
Uncontrolled BP	–	–	0.27	0.00	0.18	0.00
Age						
18–44 years	Ref					
45–59 years	–	–	1.00	0.98	1.17	0.17
≥60 years	–	–	0.92	0.39	1.05	0.74
Proportion of follow-up phone calls						
Not connected	Ref					
Connected (0.1–0.5)	–	–	0.92	0.40	0.69	0.05
Connected (0.5–0.9)	–	–	1.19	0.17	NA	NA
Connected (1.0)	–	–	0.92	0.44	1.30	0.04
Proportion of follow-up visits						
Followed up ≤0.5	Ref					
Followed up (0.6–0.9)	–	–	1.09	0.37	1.32	0.03
Followed up (1.0)	–	–	1.29	0.00	1.33	0.04
Duration of Treatment			1.07	0.00	1.11	0.00
Ward						
Lean	Ref		–	–	–	–
Intensive	2.31	0.00	–	–	–	–

^a^Odds ratios from logistic regression models adjusting for gender, voucher utilization, hub agent, provider type, BP measurement at registration, age, proportion of follow-up phone calls, proportion of follow-up visits, duration of treatment, and ward.

## Discussion

The Mumbai Hypertension Project tested a standard approach to hypertension control in over 13,000 hypertensive patients seeking medical care in private-sector clinics located in the slums of Mumbai. Several components of the intervention design—baseline and adaptive measures—led to improved patient follow-up rates over the project period and superior follow-up rates in the intensive intervention ward compared with the lean intervention ward. Patients managed by private sector providers in the intensive ward were more than twice as likely to reach BP control after 3–6 months compared with lean ward patients, but this result does not necessarily imply the intensive intervention was more effective given the higher baseline BP control rates in the intensive ward and non-randomized study design. Indeed, compared with baseline control, control overall at up to 6 months of follow-up did not improve appreciably in either study arm. Among approximately half of the enrolled patients who followed up at least once after registration, BP control increased on average by over ten percentage points compared with baseline; those in the intensive ward achieved the target BP on average 2 months sooner.

Several interventions succeeded in improving the likelihood that patients seen in the private sector would follow-up after enrollment and treatment initiation. Patients who received a follow-up reminder call were more than twice as likely to follow-up with their health care provider, and receipt of drug vouchers for free antihypertensive medications increased follow-up rates three-fold [17]. This is similar to findings reported in a study from Tamil Nadu in which telephone calls by pharmacists led to a significant increase in patient compliance with antihypertensive medications [18]. Another cross-sectional study conducted in Kolkata slums showed higher adherence rates among patients provided free antihypertensive medications [19]. The placement of hub agents was also an important driver of better follow-up rates. A study on perspectives of private providers in the state of Madhya Pradesh revealed that an increase in the number of staff for patient follow-ups, employee incentives and provision of free hypertension protocol drugs were some of the key actions that could facilitate  the adoption of IHCI strategies [20]. In facilities with AYUSH and MBBS providers, the follow-up rate was better as compared to MD providers, however, this may have been influenced by higher MD provider consultation charges. Despite these interventions, overall patient retention rates were low in both wards. Almost half of the enrolled patients failed to follow-up at least once across both wards. Similar high loss to follow-up rates have been reported by other studies conducted in public health facilities [5, 21].

Despite interventions targeted to improve follow-up and retain patients in chronic hypertension care, loss-to-follow-up was high, and in the end, the Mumbai Hypertension Project did not demonstrate success in improving BP control overall. The program did improve BP control for those patients retained in care: average 3–6 monthly cohort BP control rate improved from 24% at baseline to 38% among those who followed up. While phone calls and drug vouchers improved follow-up rates, these interventions were not seen to lead to improved BP control. In facilities with MD and MBBS providers, BP control of patients was better as compared to AYUSH providers. As AYUSH providers had high patient follow-up rates but lower BP control rates, there is potential to explore further engagement strategies with this category of providers to improve the quality of care for BP control.

Overall high loss-to-follow-up and low cohort BP control in both the lean and intensive wards of the project may be explained by the fact that many primary care clinics in India’s private sector are not geared to deliver services for hypertension and other chronic conditions. Patients seeking care in the private sector may have limited knowledge and opportunities to learn about the consequences of leaving a condition like hypertension uncontrolled, which might have led to loss-to-follow-up. Patients are also required to pay out of pocket for consultation fees and medications, which may not be financially sustainable for chronic diseases requiring life-long care. Though most services are free in the government run public sector, due to issues relating to poor accessibility, long waiting times, poor quality of care, and non-availability of doctors, many patients with hypertension prefer the private sector [22].

Our project had several limitations. Selection of the two wards compared was based on convenience, was not randomized, and as a result, the two arms were not equivalent in terms of demographic and socio-economic profiles, baseline BP control, and likely other unmeasured factors. Once the study began, a higher number of MBBS and MD providers were onboarded for the project in the intensive ward. The effect of consultation fees charged by providers on follow-up rates was likely important but not directly analyzed. The disaggregated data for previously diagnosed and new hypertensive patients were not captured. The follow-up details and the treatment history of the registered patients who were not provided or did not utilize the free drug vouchers under the project was not collected. The results come from two urban settings which might not be generalizable to other contexts. The COVID-19 pandemic further affected and interfered with project interventions and follow-up call services in the last quarter of the study.

## Conclusion

In conclusion, despite multiple project interventions to improve cohort hypertension control and retention in care, loss-to-follow-up remained high, and BP control rates improved over time only among those who followed up. To reach India’s national hypertension control goals, the private sector must be engaged by training private sector health care providers in standard IHCI treatment practices, providing chronic care financial incentives to these providers (e.g., from insurance reimbursements), and retaining patients in care with good quality and affordable services and medications.

The Mumbai Hypertension Project also demonstrated the feasibility of implementing a standard package of chronic hypertension care services among private sector service providers in two wards of Mumbai. The private sector clinics are not currently designed for chronic hypertension treatment delivery, and despite the efforts of the program, it was difficult to overcome the systematic obstacles, especially the lack of provider incentives to provide follow-up care and patient financial and convenience barriers.

## Summary

### What is known about the topic?

- In 2017, the Ministry of Health and Family Welfare, Government of India, adopted a national action plan for prevention and control of non-communicable diseases (NCD) with an aim to achieve 25% reduction in mortality from cardiovascular diseases and 25% reduction in hypertension prevalence by 2025.
- Since 70% of total outpatient care in India is provided by the private health sector, it is important to engage private sector providers in order to achieve the country’s NCD goals.

### What does this study add?

- The standard India Hypertension Control Initiative’s approach of providing (1) simple drug- and dose treatment protocol, (2) uninterrupted supply of drugs, (3) team-based care and task sharing, (4) patient-centered services by enabling availability of blood pressure (BP) monitoring and drug refills closer to the patients, and (5) monitoring system to track individual patients’ treatment and BP control was implemented in private sector clinics in the slums of Mumbai. Over the 14-month program, loss to follow-up remained high, and BP control rates improved in patients who followed up, but did not improve overall.
- In India, private sector clinics are currently not designed for chronic hypertension treatment delivery. In particular, the lack of systems and financial incentives to retain patients on treatment challenges efforts to treat and control hypertension.

## Supplementary information

Supplementary material

## Data Availability

All data generated or analyzed during this study are included in this published article and its Supplementary Information files.
