# Supplementary material for: Delivering hypertension care in private-sector clinics of urban slum areas of India: the Mumbai Hypertension Project
Source: J Hum Hypertens. 2022 Sep 24;37(9):767–74. doi: 10.1038/s41371-022-00754-1 (PMC9510164; doi:10.1038/s41371-022-00754-1)

**Supplementary material**

**Supplementary Figure 1: Map showing provider engagement in lean and intensive ward**


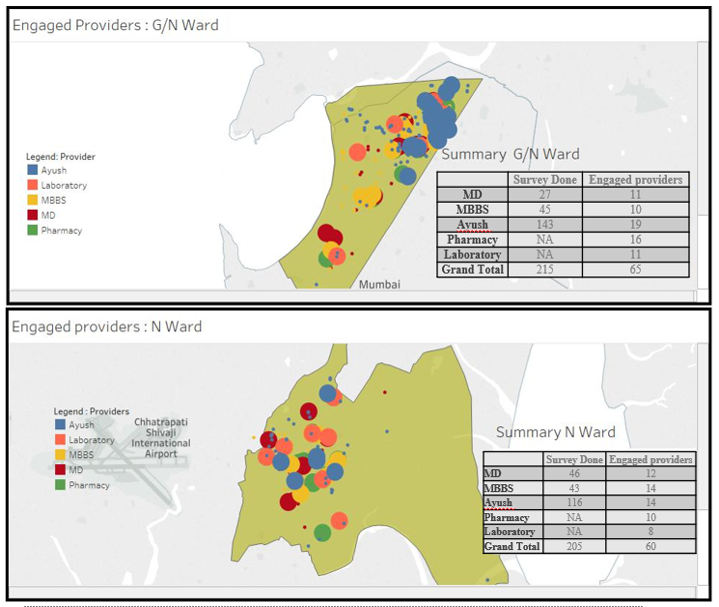


**Supplementary Figure 2: Distribution by type of providers in lean and intensive ward**

**Supplementary Figure 3: Follow-up activities with empanelled private providers in the Mumbai hypertension project**


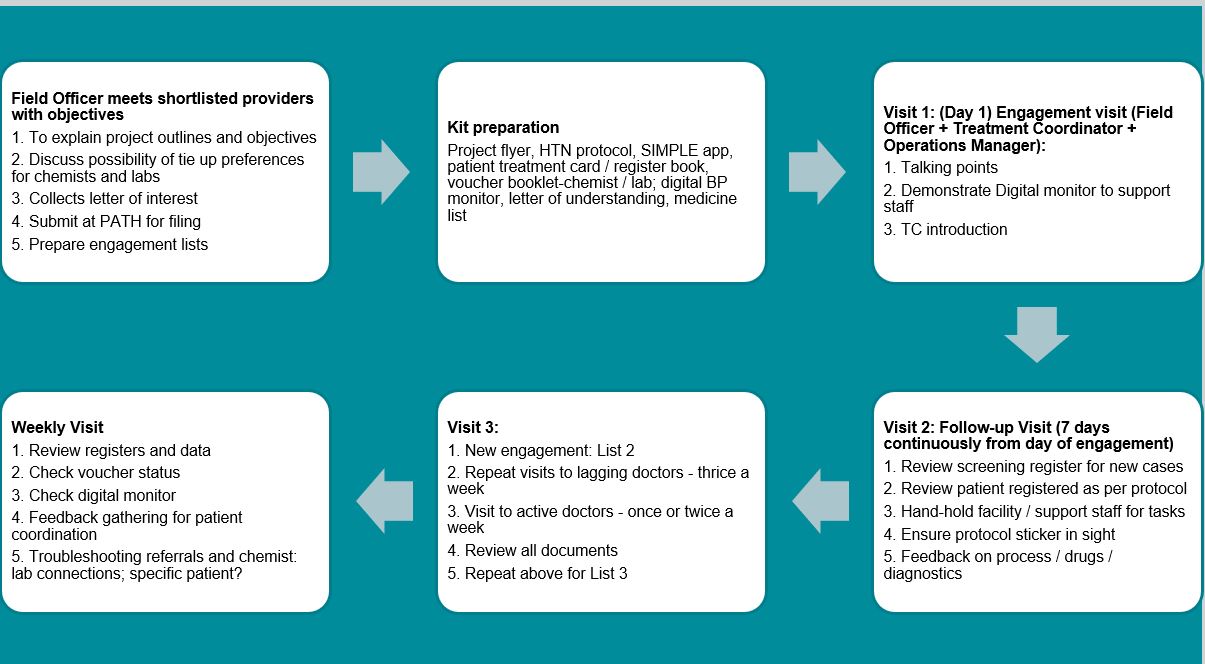


**Supplementary Figure 4: Mid-course correction strategies and operational changes**


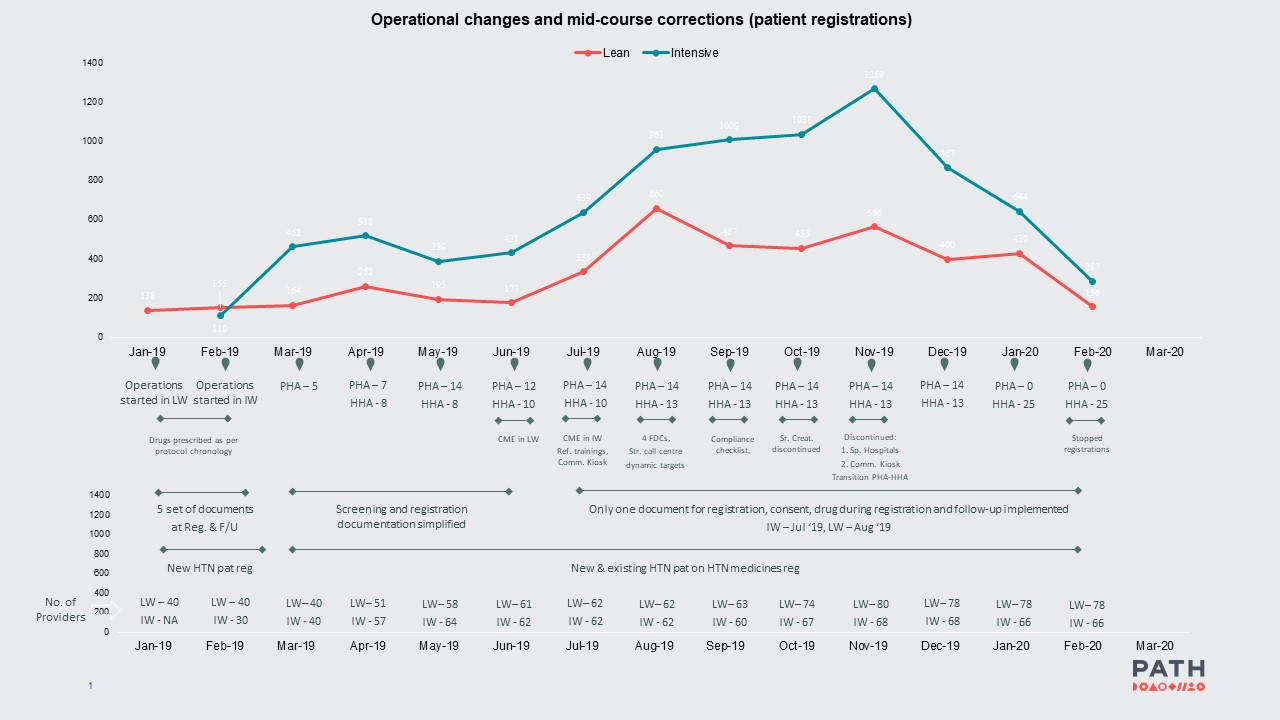


**Supplementary Figure 5: Follow up details of 13,184 registered patients in MHP**


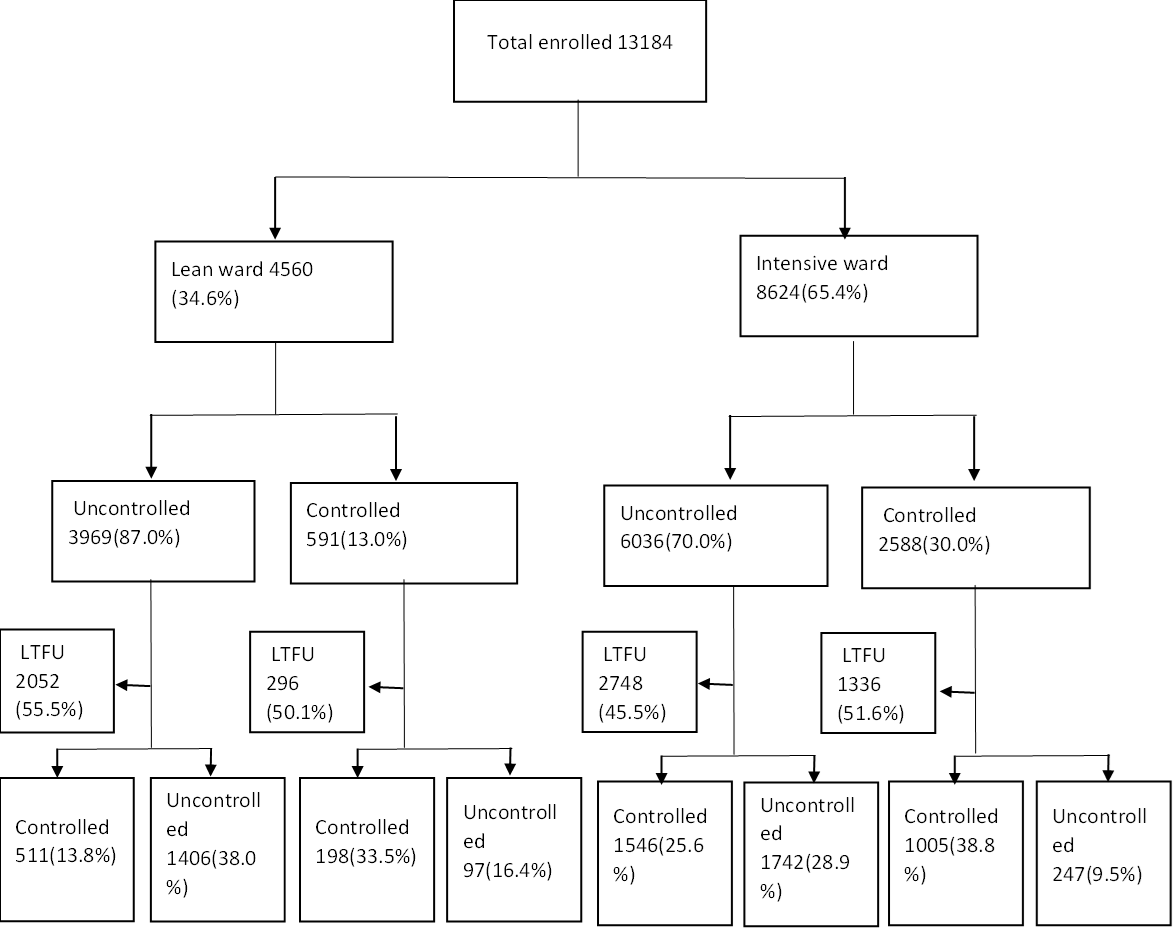

Supplement: Supplementary file 1 — Supplementary material [file 41371_2022_754_MOESM1_ESM.docx]
